# Supplementary material for: Thalamic bursts modulate cortical synchrony locally to switch between states of global functional connectivity in a cognitive task
Source: PLoS Comput Biol. 2022 Mar 9;18(3):e1009407. doi: 10.1371/journal.pcbi.1009407 (PMC8936493; doi:10.1371/journal.pcbi.1009407)
Supplement: S1 Table — (PDF) [file pcbi.1009407.s001.pdf]

Supplementary Table 1. *Parameters of DE1220 algorithm on each island.*

Island IDs	Mutation variants allowed	Adaptation scheme for parameters *F* and *C*
1	best/1/exp; rand-to-best/1/exp; best/1/bin; rand-to-best/1/bin	jDE
2	rand-to-current/2/exp; rand-to-current/2/bin	jDE
3	rand-to-current/2/exp; rand-to-current/2/bin	iDE
4	best/1/exp; rand-to-best/1/exp; best/1/bin; rand-to-best/1/bin	iDE
5	rand/1/exp; rand/1/bin	jDE
6	rand-to-current/2/exp; rand-to-current/2/bin	jDE
7	rand-to-current/2/exp; rand-to-current/2/bin	iDE
8	best/1/exp; rand-to-best/1/exp; best/1/bin; rand-to-best/1/bin	jDE
9	best/1/exp; rand-to-best/1/exp; best/1/bin; rand-to-best/1/bin	iDE
10	rand/1/exp; rand/1/bin	jDE
