# Supplementary figures and images for: Thalamic bursts modulate cortical synchrony locally to switch between states of global functional connectivity in a cognitive task
Source: PLoS Comput Biol. 2022 Mar 9;18(3):e1009407. doi: 10.1371/journal.pcbi.1009407 (PMC8936493; doi:10.1371/journal.pcbi.1009407)

Resting state coordination dynamics

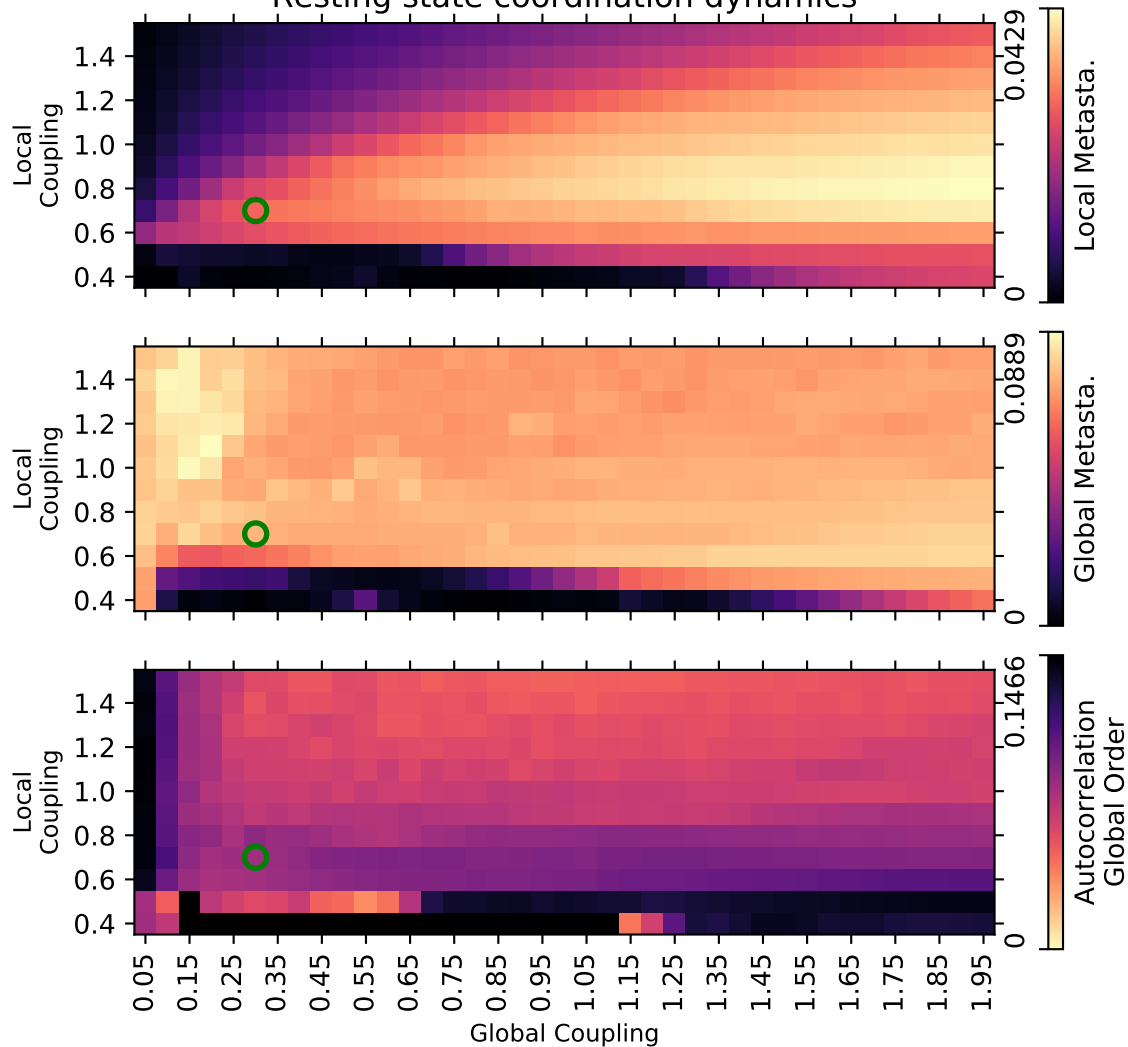

Supplement: S1 Fig — The green dot indicates the parametrization of the model. The location of the green dot was based on the idea that resting state dynamics should have simultaneously the lightest color in the three panels and the weakest coupling parameters. (PDF) [file pcbi.1009407.s002.pdf]

## Evolution: Island 1, Stage 4

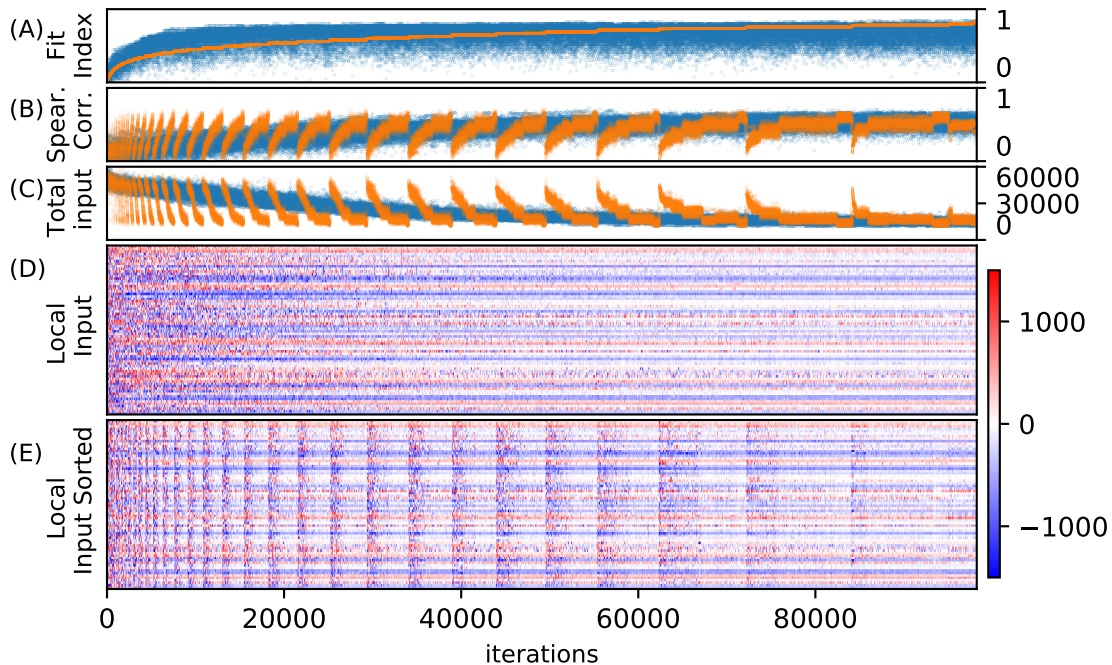

Supplement: S2 Fig — (top) Modulus of the global KOP. (middle) Modulus of the local Kuramoto order parameter (KOP) for the cortical 68 ROIs. (bottom) Temporal evolution of simulated-to-MEG fitness of within-stage dpFC for the four cognitive stages. This is similar to Fig 2B but for a much longer period of time. The MEG within-stage dpFC of each stage (Fig 1B) were compared (Eq 5) with the simulated dpFC sample-by-sample (Eq 2). (PDF) [file pcbi.1009407.s003.pdf]

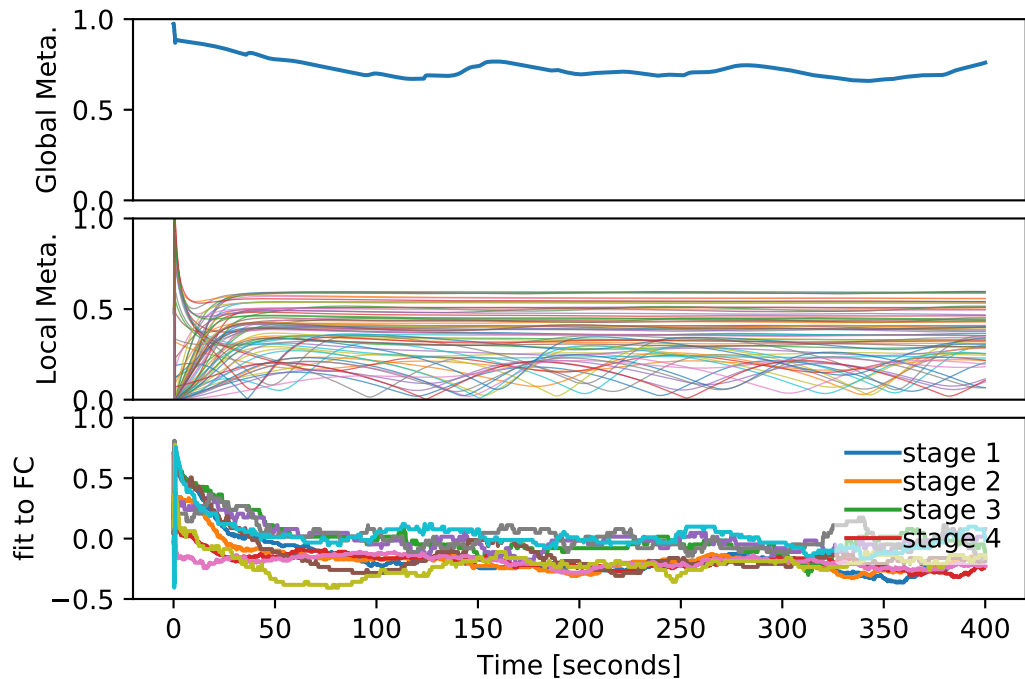

Supplement: S3 Fig — (A) fitness index f. (B) Spearman correlation, objective f1. (C) Sum of the absolute change in local coupling at the onset of the stage. Blue dots are the A, B and C values in the order that they were evaluated along the optimization process. Orange dots are the same values but sorted by the Fit Index (A). (D) Change in local coupling (thalamic input) at the onset that produces the blue dots in A, B, C. (E) Same as (D) but sorted by their Fit Index. (PDF) [file pcbi.1009407.s004.pdf]

mean and standard error of the mean

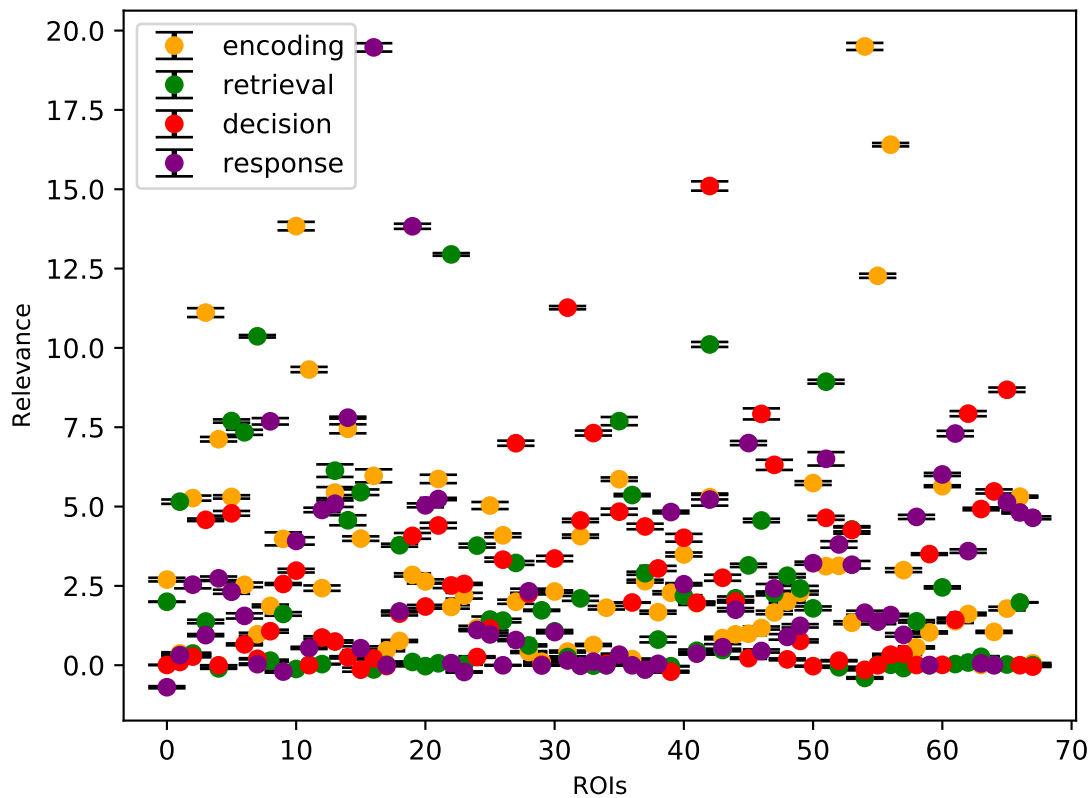

Supplement: S4 Fig — Average values are obtained for the 1,000 GWBM randomly picked from the 10,000 GWBMs with the best fitness index. (PDF) [file pcbi.1009407.s005.pdf]
