# Supplementary material for: Thalamic bursts modulate cortical synchrony locally to switch between states of global functional connectivity in a cognitive task
Source: PLoS Comput Biol. 2022 Mar 9;18(3):e1009407. doi: 10.1371/journal.pcbi.1009407 (PMC8936493; doi:10.1371/journal.pcbi.1009407)
Supplement: Attachment — Submitted filename: resonseToReviewers_v5.rtf [file pcbi.1009407.s006.rtf]

Response to Reviewers


Reviewer #1: Thank you for inviting me to review this manuscript by Portoles and colleagues, in which the authors used a combination of sophisticated statistical modelling and a simple generative model to determine the role of the thalamus in a cognitively-demanding task. The authors demonstrated a set of impressive results, however I was concerned that some of the methodological choices may have been slightly under-constrained. I have included a number of queries to this end below that I hope will help to improve the manuscript.

• Can the authors please explain why they focused on the theta band? Were their results consistent/distinct for different frequency bands?
Thank you for your comment, and for your appreciation of our paper. Indeed, this manuscript focuses only on theta band and did not consider other frequency bands. The reason for this is the following. Previously, we have shown that the phase-lag index measuring synchrony between cortical regions changes across processing stages for alpha and theta bands [1]. In the same study, we also analyzed beta band activity, however, we did not find stable networks of phase-lag index functional connectivity in the beta band. Furthermore, the gamma band has a broad power spectrum. In such broadband signals it is not straightforward to obtain instantaneous phase, which was the reason for not including gamma band activity.
The reason for not including alpha oscillations is related to its hypothesized relevance for cognitive function. The phase of theta oscillations has been related to the fundamental cognitive processes present in the task analyzed here such as attention, memory, control, and decision making (please see the references in the paper, lines 158-164). Relevant to the very nature of the generative whole-brain model is that local modulations of theta band activity have been hypothesized to mediate changes in long-range functional connectivity [2], and there is evidence that thalamic activity modulates cortical oscillations in the theta band [3]. In contrast, alpha band activity is thought to modulate thalamic activity by means of cortico-thalamic loops [4]. Furthermore, theta oscillations are known to modulate the activity in higher frequency bands [5,6]. Therefore, we believe that our selection of frequency bands contributes to understanding the mechanisms that coordinate not only theta band oscillations but also higher frequency bands.
This argumentation is included in the revision on lines 92-94, 171-177, and 403-406.

• Why was a 2s gamma filter used, but then not for the opening epoch? Were results consistent for other values of this parameter?
We apologize for the lack of clarity. In fact, a gamma distribution with shape parameter equal to two was used for all stages in a trial, from the first to the last stage. The original paper by Anderson et al 2016 [7] proposes to fix the shape parameter of the gamma probability distribution to two as “Gamma-2 distributions have the sort of shape for a temporal distribution with a long right tail, the small shape parameter still allows for a wide range of durations on individual trials.” This was reinforced by experiments reported in Anderson et al. 2016 [7] in which they let the shape parameter to be estimated by the HSMM algorithm and found that the best model also converged to a shape parameter equal to two. Furthermore, the Anderson et al. paper used synthetic data to support the assumption of fixing the shape parameter to two. The same assumption has been made by other studies that made use of the HsMM-MVPA model, e.g. [7–10] . As the goal of our study is not to assess the assumptions underlying the HsMM-MVPA we did not try other values of the shape parameter.
We have added the reason for this assumption to the manuscript (lines 477-481).

• How do the authors determine the number of stages in the HMM? Was this value consistent across individuals/trials? Could there have been any effect of learning on this parameter?
This is an important issue in HsMM-MVPA modeling. As in previous studies, we kept the number of stages constant for all subjects and trials. We do not think that there is an effect of learning that can increase or reduce the number of stages over time. The learning phase – study of word pairs – happened before the MEG data was collected. While the MEG data was collected the subjects only had to retrieve the previously learned items from memory. This is consistent with a previous computational model of associative memory [7,11]. 
To determine the number of stages we ran HsMM-MVPA models with increasing numbers of stages.  First, we performed a leave-one-subject-out cross-validation to obtain the likelihood of an HsMM-MVPA model for each subject by first fitting the model to n-1 subjects and then calculating the fit on the nth subject (that is, keeping training and testing separated). Next, we used a sign-test between an HsMM-MVPA model with M stages and an HsMM-MVPA model with M+1 stages to assess if the number of subjects better explained by an M+1 model was significantly higher than in a model with only M stages. In this way, the final model selected – a 5 stage model – represents the simplest model (i.e. with the fewest stages) that can generalize best over all subjects in terms of the likelihood of fitting the MEG data. Please see also van Maanen et al. (2021) [8] and Borst & Anderson (in press) [10], and lines 489-499 in the revision.
We have added this explanation to the manuscript (lines 489-499).

• Is the Kuramoto Order Parameter a useful model for signal envelope? Based on my understanding, the OP provides a time-varying estimate of coordinated phase in a set of different signals, however this synchrony is essentially orthogonal to changes in amplitude. I wouldn’t be surprised if the two did covary at times, however in my opinion, the burden of proof is on the authors to demonstrate that this assumption is in fact warranted in this data.
The Kuramoto Order Parameter has been shown to describe the aggregated dynamics of neural activity accurately in previous studies [12]. In our case, an MEG sensor records the aggregated neural activity generated by millions of neurons in a cortical region. Each active neuron in this region generates an electric field. The measurable electric potential depends on the alignment of active neurons and the temporal synchrony of the dipole moments generated by the electric fields. The neurons that contribute to the MEG signal are parallel to each other. Therefore, the strength of the measurable electric potential in the region is proportional to the number of synchronized dipole moments [13,14]. In our model, the Kuramoto order parameter represents the synchrony of dipole moments.
We have added this explanation to the manuscript on lines 529-538.

• Was the inter-regional connectivity presumed to be ‘all-to-all’ in the Kuramoto model and if so, why was this considered to be an effective assumption?
Thank you for the question. Inter-regional connectivity was given by the density and length of the neural fibers that connect cortical regions. Density and length of neural fibers was obtained with diffusion magnetic resonance imaging. The density of fibers weighted the inter-regional coupling strength (variable A in equation 2 and 3). The length of the fiber determined the inter-regional time-delay (τ in equations 2 and 3). So, although it was all-to-all, the strength of the connections was based on anatomical data.
We have clarified this in the methods section on lines 650-651

• Minor: on P8 L74: the following statement requires a citation: “cortically projected magnetoencephalographic (MEG) recordings … have a sufficiently fine temporal resolution to measure cognitive stages”
In this study the MEG system sampled at 1 kHz, while processing stages last for a minimum of about 100 milliseconds and theta band activity has a maximum frequency of 8 Hz. See for a similar assumption, Anderson et al., 2018, Psychological Science [9].We added this citation to the statement.

Reviewer #2: This was an exciting, albeit difficult, paper to read. In essence, the paper deals with the question of how cortical activity can be sequenced in functional, coordinated stages of processing and how these stages can be properly sequenced. The manuscript attempts to answer this question using a two-pronged approach: First, they use an innovative technique (HSMM-MVPA) to identify the most reasonable number of cognitive stages that make up a simple associative memory task. These stages are characterized by extended periods of stable MEG activity and separated by quick “bumps” in which the topological landscape of MEG activity quickly changes. Second, the authors characterized the functional connectivity (FC, measured as phase coherence) between 5,000 cortical nodes in the theta band, and showed that the stages thus identified are characterized by remarkably different FC patterns that are consistent with their putative functional roles. Finally, the authors explore what could be the origin of the transition between stages. Building upon previous influential models of the Basal Ganglia, they suggest that stage transitions could be triggered by short bursts of basal-ganglia-directed thalamic bursts. They simulate the effects of such bursts using a Global Whole-Brain Modeling approach using Kuramoto oscillators (Very cool!) and produce simulated FC data that look remarkably similar to the empirical data.

This paper is a beautiful tour de force and a great example of sophisticated, interdisciplinary approach to neuroscience; I thoroughly enjoyed reading it. My main comments concern its difficulty, novelty, and conclusions, and are detailed below.
We are very pleased to hear that you enjoyed that manuscript and that you appreciate the effort behind this manuscript. Just to be sure, in fact the FC was measured over 68 ROIs and not over the 5,124 dipoles used to compute sources of MEG data. These ROIs were computed by applying the Desikan-Kelliany atlas [15] to the 5,124 dipoles. We have gone through the entire paper to make sure that this is clear.  

1. DIFFICULTY. The manuscript is very difficult to read, even for readers who are familiar with the analysis/modeling techniques detailed in this paper. This problem is compounded by the PLOS Comp Bio format, which requires methods at the end and some details in the supplementary materials. I have detailed some of the details I still could not find answers to at the end of this review, in a a special, fourth “Clarifications” section below. The bottom line is that the MEG analysis, although clear, is hard to connect piece-by-piece to the model part; that the figures are beautiful but incredibly dense to read; and that some technical jargon leaves some details necessarily unspecified.
Indeed, the PLOS Comp Bio format does not always make it easy to follow a paper, in particular with complex models. We would like to thank you for pointing out the difficulties in understanding below, we think that addressing those issues have improved the paper considerably.


2. NOVELTY. Although this paper is brilliant, it is built on data and models that had been previously obtained by the authors and already published elsewhere---one of their papers, in fact, seems to be still unpublished and available only as a preprint. To understand this paper, I had to download and read the others. Thus, the net result is that this paper is like the tip of an iceberg, and the full project requires having knowledge of the already-done research to fully appreciate it. Importantly, most of the novelty part is built in these other papers. When this is accounted for, the net contribution of this paper seems to be the addition of BG-modulated bursts to the GWBM and the focus on theta FC in the stages. This is definitely sufficient to motivate a stand-alone paper but it should be stressed a bit more in the paper and abstract. Similarly, some of the conclusions in this paper are relevant to the HSMM analysis (already published) but not necessarily to this paper specifically. For example “[line 379] our results suggest that the decision is made and evaluated in the last two stages. We interpreted the penultimate stage as a decision process in which memories are transferred to parietal areas by coordinating left-temporal regions with parietal regions, mediated by local frontal and fronto-parietal coordination”. I also agree with the conclusion that one important aspect of the HSMM models is that they get rid of trial variability in interpreting the sources of activity (there is still uncertainty about which stages need to be identified, and this algorithm, although amazing, is not a consensus yet). However, rather than a conclusion, this seems like a finding that has been established in the authors’ previous papers and that works as a foundation for this paper, so it should be highlighted at the beginning, not at the end.

We really appreciate that you went back to read our previous papers. Indeed, this project builds on the previous papers (the pre-print should be published soon) as they were necessary to develop and demonstrate the validity of some of the methods of this paper. Nonetheless, we think that the contribution of this paper goes beyond the BG-modulated burst. Here, we provide a new way to analyze MEG data by time-locking the data to the onset of cognitive processes and combining that with functional connectivity measures. This analysis made it possible, for example, to reach the conclusion in line 379 that you mention. Furthermore, we think that another novel contribution of this study is to show that a dpFC network emerges at the onset of each cognitive stage. What we did not show in our previous paper in Eur J Neurosci is that this dpFC network remains stable until the end of the cognitive stage and then disappears. Although it seems intuitive that each cognitive function requires a particular interaction of neural activity, such phenomena had not been measured before.
Another novelty of this study is that we built the first GWBM that can simulate data of a cognitive task, in contrast to merely resting-state data, which is the focus of previous GWBM studies. Previously, simulating such task data remained elusive as there are numerous challenges. Here we solved some of these, such as the initialization of the model variables (i.e. phases and amplitudes), the optimization of the parameters, and the definition of a suitable fitness function, and modeling the transition between cognitive processes.
In the revision, we tried to clarify the specific contributions of the current study.

. CONCLUSIONS. To a certain extent, some of the main conclusions are a bit overstated. Crucially, if I am reading the modeling part correctly (and I might not---see my first point), the short bursts of BG-Thalamic are not simulated directly by just approximated as transient increases in the L parameter. If this is the case, then what kind of evidence do we have that these increases come from the BG circuit and not some other cortical circuit? Importantly, the simulated bursts seem to affect all possible cortical nodes, but NOT all cortical regions receive BG projections. Anatomically, their projections are restricted to the frontal regions (and certainly not all frontal regions equally). Some of the conclusions of the paper suggest that some frontal regions can facilitate other regions transition between stages; however, it is not clear whether this is implemented in the model. Also, it is not possible to conclude that these results corroborate “[line 356] the hypothesis that the local modulations of phase synchrony in the theta-band mark a change in long-range functional connectivity and enable a new cognitive function”. Of course, the results are *consistent* with this hypothesis, but the results simulated only theta activity and the empirical results also filtered out other bands. So, the conclusion might be more bland: Burst that are believed to simulate BGT bursts of activity between stages produce similar changes in theta activity.
This an important point: we certainly did not want to overstate the conclusions and indeed did not include the basal ganglia-thalamus complex explicitly in our model. First, to clarify a relevant part of the model, the short bursts of activity are assumed to come from the thalamus, not the basal ganglia. In cognitive theories the basal ganglia is thought to drive the thalamus which then modulates cortical activity [16,17] at the onset of cognitive processes. Therefore, we did not explicitly include the BG as it projects to the thalamus and not to the cortex [18]. The pulsatile increase or decrease of the parameter L represents short and transient modulation of local excitation or inhibition [19] as there is evidence suggesting that bursts of thalamic activity are targeted to either excitatory or inhibitory cortical synapses to coordinate information transfer between cortical region during behavioral tasks [20–23]. Nevertheless, we agree with the reviewer that there could be other brain regions involved in modulating local cortical activity with pulse-like events or another mechanisms.For this reason, we had included on lines 350 to 353 of the discussion the following “Additionally, we have assumed that perturbations of cortical dynamics at the onset of cognitive stages come exclusively from the thalamus. However, there might be other regions such as the hypothalamus that modulate cortical activity in the same or another way that is not included in our model.”
Our results shows that some frontal regions are very relevant to enable dpFC between other regions.  Interestingly, there was not any specific addition to the model to account for this phenomenon. Such results appeared naturally as a consequence of the interaction between the neural dynamics, the pulses, and the anatomical connectivity between regions.
In the new version of the manuscript, we have moderated our statements as you suggested and we have tried to clarify the points that have generated confusion.


4. SPECIFIC CLARIFICATIONS.

4.1. The dpFC equation first appears in line 465 because of the PLOS Comp Bio format; however in the main section some aspects of dpFC should be immediately introduced. A brief overview of how are the phase angles calculated and a clarification of that FC is calculated instantaneously at every sample is important to understand Figure 1C. This is crucial because there are several ways to calculate phase-based measures of FC, some of which are segment based or trial-based (e.g. phase of max coherence) while others are instantaneous, like the one described.
Thank you for bringing this up, as it is a very relevant point that should be clear. We have rewritten parts of the results and methods sections to clarify it.

4.2. Still, in the dpFC equation on line 465, I am not sure I understand what 465 the subscript “nt” in “Im(S^ij_nt)” represents.
We appreciate your careful attention to details. We have divided Eq. 1 into two equations (equations 1 and 2). Eq. 1 contains the formula of within-stage dpFC with new indexes, and Eq. 2 contains the formula of across-trial dpFC.    

4.3. Figure 1 is very hard to parse, although I admit it is one of the most beautiful figures I have ever seen). Specifically: Do the lines in (C) represent the *mean* FC across all links? We do have over 25,000K links, and it seems like it would be impossible to plot them all in a single brain figure. Also, I still do not understand the exact meaning of the magenta lines in panel (D).
Thank you. Yes, the colored lines in Figure 1C are the average over some of the links between the 68 nodes. Specifically, the colored lines are the average of the links that had a significant within-stage dpFC for the same stage. These are the links shown in Figure 1b. The gray color lines are the average of all the links that had not a significant within-stage dpFC for the same stage (i.e. the links not included in Figure 1B).
The magenta lines define the boundaries of the two time windows that were used to measure the relative change in local coordination at the onset of a cognitive stage. The first window spans from -60 to -10 milliseconds from the onset of the stage, while the second window spans from 0 to 50 millisecond after the onset of the stage.
We have extended the description in the legend of Figure 1 to clarify both points. 

4.4. Line 167: “The significance of dpFC was obtained using 200 surrogate data sets with random circular shifts of the original phases” Do you mean, you did some form of phase permutation test?
Yes. It is similar to a permutation test that changes the temporal relationship between two phases, while it keeps the structure of the phase. We used the function circshift from Matlab.  It is like if you join the end and the beginning of the signal’s phase in a trial in such a way that the signal now forms a closed circle. Next the signal is rotated clock-wise by a random number of degrees or radians. Then, dpFC is computed between the “rotated” signal and another non-rotated signal, and we assessed whether the empirically-observed signal is larger than these 200 surrogate signals.
We have added an intuition for what the circular shift does to the data in the manuscript.

4.5. Line 166 says that this was calculated only for theta band. How did you remove the other frequencies?
We removed the other frequencies by bandpass filtering. Specifically, the following information is included in the methods section in  lines 502-507: “The parcellated MEG data were bandpass filtered (cut-off frequencies: 3.8, and 8.5 Hz; forward-backward IIR Butterworth filter of order 4) and epoched from -0.4 seconds before stimulus onset to 0.4 seconds after the response.”

4.6. Line 185: “Local synchrony was operationalized as the envelope of the theta band analytic signals in each” What is that??
The analytic signal is the same signal divided into its real and imaginary components which can be transformed into the phase and the envelope the signal. This was explained in the methods section: “Epochs were Hilbert-transformed to the analytic signal using a symmetric padding of 0.4 seconds to avoid edge artifacts.”
We now added a clarification in the methods section. 

4.7. Line 256: “All model results were computed from 1000 models randomly selected from the top one percentile of models after the optimization.” I assume the top 1% models are ranked on the bases of fit? If so, is there any guarantee that the top 1% models would cover similar parameter spaces? And, if not, what would be the implications?
Thank you for raising this point. Indeed, it could happen that the top 1% of the models cover different areas of the parameter space. Stochastic optimizers, like the evolutionary optimizers used here, do not guarantee converging to a global optimum. This is the very reason why we did not take only the best optimization result but 1000 models randomly selected among the 10,000 models with the best fitness index (~top 1%). We took these 1000 models to be able to derive robust and general conclusions about the model and the phenomenon that it describes, rather than specific about a particular parametrization of the model. Nonetheless, the parameter space of the 1,000 models is relatively small. This is partially visible in the Figure S3, and the small standard error of the mean on all results. Furthermore, we performed other sanity checks. For example, the figure below shows the mean relevance of a region (the size of the nodes in figure 2) and mean standard error across the 1.000 models. The mean standard error is very small. This figure shows that irrespectively of the parameters on each of the 1,000 models, the relevance of a region is consistent as the small standard errors of the mean show.
We expected that several parameterizations of the GWBM could simulate MEG data similarly, as in our previous study of resting state MEG data. There we gave three interpretations of the results. First, the brain might have the flexibility to produce similar patterns of cortex-wide functional connectivity under different local properties. Second, the optimizers were not able to find a single global optimum. Third, the fitness function of the optimizers did not have enough information. Eventually in the resting state study we pursued the last interpretation. The resting state study showed that although simulated functional connectivity was remarkably similar between two different parametrizations of the GWBM, the dynamics of local synchrony were diametrically opposed. Therefore, in the current paper we included information about local synchrony in the fitness function (f_2_ in the fitness index). Furthermore, here we have designed a more restrictive fitness function which focusses more on significant links of directed functional connectivity than on a full symmetric functional connectivity matrix as it is common on resting state studies.
We added this figure to the supplementary information with references in the manuscript.


4.8. I am not sure (Figure 2) if the GWBM model actually models every node, or only those significant, or only the significant links, or just some average. I believe that the GWBM models every node and every link (5,000 nodes and 5,000^2 links), but I might be wrong. This line of the text makes it difficult to understand at what level the model predictions were drawn: “[265] The fitness was quantified as the proportion of links with the same phase-lag direction as in the MEG data” The proportion of links does not seem to be a measure or a single lin, but just for a stage or maybe a node.
The GWBM had only 68 nodes. MEG sources were modeled with ~5.000 dipoles but then we aggregated the activity within each of the 68 cortical regions defined by the Desikan-Kelliany atlas [15]. Therefore, there were only 68 signals of aggregated MEG source activity and 68 nodes in the GWBM.
We have clarified this very relevant point in the manuscript on lines 149-152

4.9. From the GWBM model description, I could not understand whether each bursts for each region was modeled separately. This seems important, and I might be just too dense to understand it after multiple readings, but it would be great if it could be highlighted.
Thank you for the question. At the onset of each stage there were 68 simultaneous bursts, one burst for each node in the GWBM.
We have clarified this point in the manuscript.

4.10. Very Minor Comment: There is a typo in abstract “T o test” → “To test”
Thank you.


References


1. 	Portoles O, Borst JP, van Vugt MK. Characterizing synchrony patterns across cognitive task stages of associative recognition memory. Eur J Neurosci. 2018;48: 2759–2769. doi:10.1111/ejn.13817
2. 	Voloh B, Womelsdorf T. A Role of Phase-Resetting in Coordinating Large Scale Neural Networks During Attention and Goal-Directed Behavior. Front Syst Neurosci. 2016;10. doi:10.3389/fnsys.2016.00018
3. 	Ketz NA, Jensen O, O’Reilly RC. Thalamic pathways underlying prefrontal cortex-medial temporal lobe oscillatory interactions. Trends Neurosci. 2015;38: 3–12. doi:10.1016/j.tins.2014.09.007
4. 	Halgren M, Ulbert I, Bastuji H, Fabó D, Erőss L, Rey M, et al. The generation and propagation of the human alpha rhythm. Proc Natl Acad Sci USA. 2019;116: 23772–23782. doi:10.1073/pnas.1913092116
5. 	Sauseng P, Griesmayr B, Freunberger R, Klimesch W. Control mechanisms in working memory: a possible function of EEG theta oscillations. Neurosci Biobehav Rev. 2010;34: 1015–1022. doi:10.1016/j.neubiorev.2009.12.006
6. 	Canolty RT, Edwards E, Dalal SS, Soltani M, Nagarajan SS, Kirsch HE, et al. High Gamma Power Is Phase-Locked to Theta Oscillations in Human Neocortex. Science. 2006;313: 1626–1628. doi:10.1126/science.1128115
7. 	Anderson JR, Zhang Q, Borst JP, Walsh MM. The discovery of processing stages: Extension of Sternberg’s method. Psychol Rev. 2016;123: 481–509. doi:10.1037/rev0000030
8. 	van Maanen L, Portoles O, Borst JP. The Discovery and Interpretation of Evidence Accumulation Stages. Computational Brain and Behavior. 2021;4: 395–415. doi:10.1007/s42113-021-00105-2
9. 	Anderson JR, Borst JP, Fincham JM, Ghuman AS, Tenison C, Zhang Q. The Common Time Course of Memory Processes Revealed. Psychol Sci. 2018;29: 1463–1474. doi:10.1177/0956797618774526
10. 	Borst J, Anderson JR. Discovering Cognitive Stages in M/EEG Data to inform Cognitive Models. 2nd ed. B Turner (Eds), An Introduction to Model-Based Cognitive Neuroscience. 2nd ed. Springer: New York; 
11. 	Borst JP, Anderson JR. The discovery of processing stages: Analyzing EEG data with hidden semi-Markov models. NeuroImage. 2015;108: 60–73. doi:10.1016/j.neuroimage.2014.12.029
12. 	Montbrió E, Pazó D, Roxin A. Macroscopic Description for Networks of Spiking Neurons. Phys Rev X. 2015;5: 021028. doi:10.1103/PhysRevX.5.021028
13. 	Lopes da Silva F. EEG and MEG: Relevance to Neuroscience. Neuron. 2013;80: 1112–1128. doi:10.1016/j.neuron.2013.10.017
14. 	Buzsáki G, Anastassiou CA, Koch C. The origin of extracellular fields and currents — EEG, ECoG, LFP and spikes. Nat Rev Neurosci. 2012;13: 407–420. doi:10.1038/nrn3241
15. 	Desikan RS, Ségonne F, Fischl B, Quinn BT, Dickerson BC, Blacker D, et al. An automated labeling system for subdividing the human cerebral cortex on MRI scans into gyral based regions of interest. Neuroimage. 2006;31: 968–980. doi:10.1016/j.neuroimage.2006.01.021
16. 	Stocco A, Lebiere C, Anderson JR. Conditional routing of information to the cortex: A model of the basal ganglia’s role in cognitive coordination. Psychol Rev. 2010;117: 541–574. doi:10.1037/a0019077
17. 	Stocco A. A Biologically Plausible Action Selection System for Cognitive Architectures: Implications of Basal Ganglia Anatomy for Learning and Decision-Making Models. Cogn Sci. 2018;42: 457–490. doi:10.1111/cogs.12506
18. 	Stewart TC, Bekolay T, Eliasmith C. Learning to Select Actions with Spiking Neurons in the Basal Ganglia. Front Neurosci. 2012;6. doi:10.3389/fnins.2012.00002
19. 	Maistrenko YL, Lysyansky B, Hauptmann C, Burylko O, Tass PA. Multistability in the Kuramoto model with synaptic plasticity. Phys Rev E. 2007;75: 066207. doi:10.1103/PhysRevE.75.066207
20. 	Malekmohammadi M, Elias WJ, Pouratian N. Human Thalamus Regulates Cortical Activity via Spatially Speciﬁc and Structurally Constrained Phase-Amplitude Coupling. : 11. 
21. 	Saalmann YB, Pinsk MA, Wang L, Li X, Kastner S. The Pulvinar Regulates Information Transmission Between Cortical Areas Based on Attention Demands. Science. 2012;337: 753–756. doi:10.1126/science.1223082
22. 	Ramcharan EJ, Gnadt JW, Sherman SM. Higher-order thalamic relays burst more than first-order relays. Proc Natl Acad Sci USA. 2005;102: 12236–12241. doi:10.1073/pnas.0502843102
23. 	Kloc M, Maffei A. Target-Specific Properties of Thalamocortical Synapses onto Layer 4 of Mouse Primary Visual Cortex. J Neurosci. 2014;34: 15455–15465. doi:10.1523/JNEUROSCI.2595-14.2014
